# The healthcare system in Haiti

**DOI:** 10.3389/fpubh.2025.1603076

**Published:** 2025-12-17

**Authors:** Catiana Dorilien, Clara Aleida Prada Sanabria

**Affiliations:** Graduate Program in Public Health, Department of Health, State University of Feira de Santana, Feira de Santana, Brazil

**Keywords:** Primary Health Care, health, healthcare system, Haiti, universal coverage

## Abstract

The Haitian healthcare system is characterized by numerous challenges, including a strong dependence on external aid, inequalities in access to primary care, insufficient infrastructure, and limited public funding, which undermine the goal of universal health coverage in the country. This article aims to describe the general characteristics of Haiti's healthcare system, focusing on the adopted healthcare model, organizational structure, human resources, system organization, and management. Documentary research was conducted, including archives, scientific articles, and reports from governmental, non-governmental, and international organizations. Additionally, websites of various organizations and research institutes, notably the Ministry of Health and Population and the World Bank, were consulted. The study's findings highlight several critical issues within Haiti's healthcare system, such as inadequate infrastructure, a shortage of healthcare professionals, and insufficient funding. Only 19.28% of the population has access to Primary Health Care, highlighting limited basic services, especially in remote areas. Dependence on external aid and the private sector undermines equity and coverage, stressing the need for strategic measures. This study contributes to the global literature on fragile health systems by illustrating how donor dependence and governance weaknesses interact to shape healthcare outcomes. It also brings novelty by mapping the contradictions between the Ministry of Health and Population's policies and field practices in the context of aid dependency.

## Introduction

The healthcare system is an interconnected set of factors that influence the health and wellbeing of populations, including biological, behavioral, environmental, and organizational aspects (1). According to the Ministry of Public Health and Population (2), it is structured to ensure governance, resource creation, financing, and service delivery. However, each country faces unique challenges and characteristics.

In the United States, the healthcare system is private and market-based (3). In contrast, Brazil's Unified Health System (SUS) is universal and public, supplemented by the private sector. Data from (4) indicate that 24.6% of the Brazilian population has private health insurance, characterizing a mixed system. The organization of health systems varies according to historical and political contexts. In the United States, the system remains dominated by private insurance, leading to significant inequalities in access, while in Brazil, the establishment of the Unified Health System (SUS) has enshrined the principle of universal coverage despite persistent challenges in financing and governance. These international experiences provide contrasting reference points, but the case of Haiti presents major specificities, notably a strong dependence on international aid, a low share of public funding, and a fragmentation of services.

In Haiti, the system aims to provide universal health insurance, eliminating financial barriers to access (5). However, many still face economic and geographical challenges in obtaining healthcare services (6). Additionally, there is a lack of clear mechanisms for managing public and private healthcare resources. The Haitian system consists of a contributory model for those who can afford it and a non-contributory model funded through taxes and international aid (Ibid.).

According to Article 22 of the 1987 Haitian Constitution, the state recognizes the right to housing, education, and social security but does not explicitly mention the right to health. Although health is part of social security, the protection system faces significant challenges. The 2016–2017 Haiti Mortality, Morbidity, and Service Utilization Survey (EMMUS VI) reported that only 5% of men and 3% of women had health insurance, totaling less than 10% of the population (7). Recent data (8) indicate that coverage remained stagnant between 2016 and, 2021 with only 10 insurers covering less than 10% of the population. The Haitian health insurance system includes both public and private sectors.

The *Office d'Assurance Accidents du Travail, Maladie et Maternité* (OFATMA) is the only public health insurer, providing mandatory coverage for public sector employees and formal private sector workers, yet covering only 2% of the population (8). Nine private insurers offer individual health plans for employees and their dependents, covering approximately 4% of the population (Ibid.).

Data from EMMUS VI and (8) indicate that universal coverage in Haiti remains an aspiration, particularly in rural areas. As (6) highlights, in a country where 70% of the population is unemployed (9) restricting coverage to formal workers poses a major barrier to implementing a universal system. Only 2.1% of the rural population and 14.4% of the urban population have health insurance.

According to (10), health policy refers to the actions or inactions of the state in addressing health issues and their social, environmental, and cultural determinants, as well as the regulation of goods and services that affect the population. This perspective broadens the understanding of health by incorporating structural factors. Paim argues that health policy extends beyond traditional care and curative actions, encompassing state measures to promote equity and regulate factors influencing public health. When the state ensures adequate living conditions and healthcare access, it engages in a beneficial “state action.” Conversely, inaction due to lack of resources, negligence, or favoritism toward private interests constitutes a “state omission,” exacerbating inequalities and harming the most vulnerable.

In Haiti, the MSPP defines health policy as a complex process involving the government, civil society, international partners, and the population. Its vision (11) aligns with Paim's definition by recognizing health as a multidimensional process that extends beyond healthcare services to include regulation and the promotion of conditions that impact society as a whole.

This article aims to describe the general characteristics of Haiti's healthcare system, including its organizational and management structure, human resources, financing, and access to healthcare. In this context, the literature highlights what Paim defines as the “omission of the State,” that is, the structural inability of public authorities to fully assume their role as guarantor of universal access to care, thereby opening space for NGOs and donors. To analyze these dynamics, this article also draws on the conceptual framework proposed by the World Health Organization (WHO), through the six building blocks of a health system: service delivery, health workforce, information, medical products and technologies, financing, and governance.

## Methodology

This qualitative case study examines Haiti's healthcare system through plans, policies, and studies from 2010 to 2024. It analyzes the healthcare model, organization, management, human resources, financing, and access. Secondary data collection was based on documents from Haiti's Ministry of Public Health and other institutions. Additional bibliographic research included archives, theses, articles, and reports from governmental and international organizations. Thematic content analysis was used as the primary method for data analysis. Data coding was carried out manually, following a three-step process: pre-analysis (in-depth reading of the selected documents), categorization (development of an open coding system based on identified themes such as centralization, external funding, health personnel, primary care, etc.), and thematic coding (systematic application of the codes to relevant excerpts from the documents).

The primary information on the organizational structure was collected from the official website of MSPP, which provides data on the organization of the country's healthcare system. Furthermore, the main details regarding the functions performed at different levels of care were found in the *Paquet Essentiel de Services* (PES), published in 2015. This document is regarded as a reference tool for healthcare institutions, whether public, private, or mixed, with its main objective being the expansion of basic healthcare service coverage for the entire population.

The source of data and information on human resources was the Strategic Development Plan for Health Human Resources 2030, published in 2017 under the presidency of Jovenel Moïse from 2017 to 2021. In the analysis of human resources, WHO criteria and the distribution by department were considered.

Data on current health expenditures per capita (% of GDP) between 2000 and 2020 in Haiti and financing information were collected from the World Bank database (12) and the CNS (2012–2013, 2016–2017, 2017–2018, and 2018–2019), respectively. Additionally, data and information on mortality, healthcare service utilization, MSPP strategies, access to care, quality of healthcare services, reproductive health, and other indicators were sourced from: EMMUS-VI 2016–2017 (2018), EPSS 2017–2018 (2018), PDS 2012–2022 (2012) and 2021–2031 (2021), PNS (2012) e PSNSSR 2019–2023 (2019).

## Results and discussion

This section presents the administrative structure and management of the Haitian health system, with an emphasis on Primary Health Care. It addresses financing, external dependency, and resource management, as well as access to care and the availability of professionals according to WHO criteria.

According to Article 61 (13) Haiti's Ministry of Public Health operates within an administrative framework of 10 Departments, 146 Communes, and 571 Communal Sections. With 11.6 million people (14), 40.7% in rural areas, the country faces high illiteracy (39%) (15), extreme poverty, and inequality (HDI 0.552, Gini 0.41) (16–18). Food insecurity remains severe, with 49% of Haitians (5.54 million) at risk in 2025 (19).

**Figure 1:**
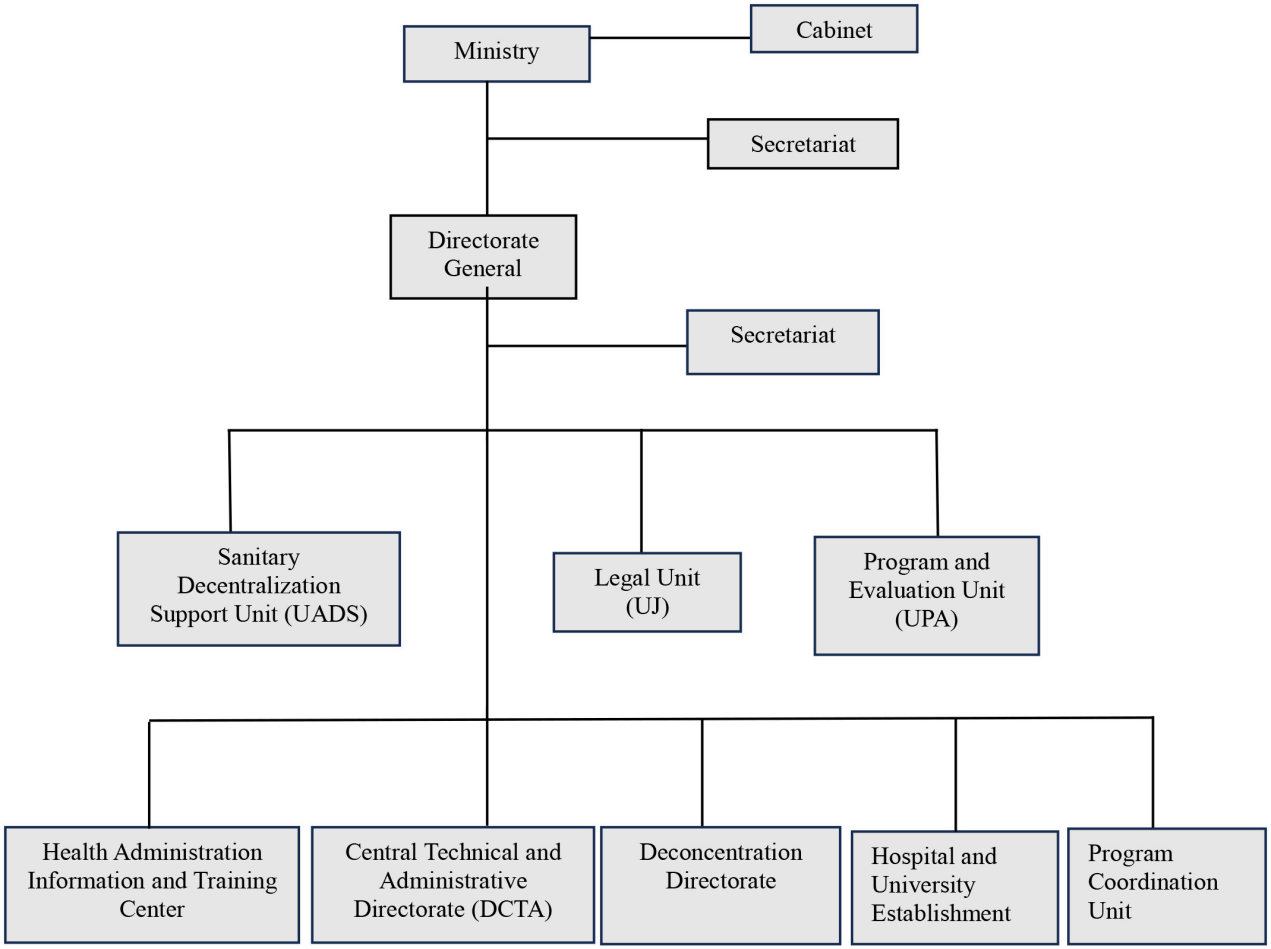
Administrative organization of the Haitian health system. Figure created by the authors based on Ref. (22).

The communal section, the smallest administrative unit, is managed by a council of three members elected for 4 year, with support from the Municipal Assembly. The commune, governed by a municipal council elected for the same term, is part of the arrondissements, which are administered by the councils of the communes that comprise them (Art. 75). The department, the largest territorial division, encompasses multiple arrondissements and is governed by a three-member council elected by the Departmental Assembly (Art. 76–78). The MSPP follows this structure, with a general directorate, as well as central and departmental directorates (Figure 1).

As the principal government body overseeing healthcare (20), the MSPP defines health policies and coordinates healthcare services. Strategic decisions are made at the national level, while central and departmental directorates oversee hospitals and health centers. Positioned at the core of the health system, the MSPP operates across three levels of complexity—primary, secondary, and tertiary—and is responsible for policy implementation, program coordination, and sector regulation (21).

In each department, there is a Departmental Health Directorate responsible for coordinating medical care (22). The group of Central Directorates consists of technical and administrative directorates, program coordination units, support units, and the Health Administration Information and Training Center (CIFAS), all reporting to the Directorate-General (22).

### Technical directorates

- Directorate of Population and Human Development (DPDH)
- Directorate of Health Services Organization (DOSS)
- Directorate of Training and Professional Development in Health Sciences (DFPSS)
- Directorate of Family Health (DSF)
- Directorate of Nursing Care (DSI)
- Directorate of Health Promotion and Environmental Protection (DPSPE)
- Directorate of Pharmacy, Medicines, and Traditional Medicine (DPM/MT)
- Directorate of Epidemiology, Laboratory, and Research (DELR)

### MSPP administrative directorate includes

- Directorate of Administration and Budget (DAB)
- Directorate of Human Resources (DRH)

### Program coordination units

- Coordination Unit for Infectious and Communicable Diseases, STDs/HIV/AIDS, Tuberculosis, Malaria, Lymphatic Filariasis, and Leprosy
- National AIDS Control Program (PNLS)
- National Malaria Control Program (PNCM)
- National Blood Transfusion Safety Program (PNST)
- National Coordination Unit for the Immunization Program
- National Coordination Unit for the Nutrition Program
- National Ambulance Center (CAN)
- National Public Health Laboratory (LNSP)
- National Health Management Unit (UNGUS)

### Support units

- Health Decentralization Support Unit (UADS)
- Evaluation and Programming Unit (UEP)
- Legal Unit (UJ)
- Communication and Public Relations Unit (UCRP)
- Unit attached to the Directorate-General
- Health Administration Information and Training Center (CIFAS).

The Haitian public health system is organized into three levels. The first level consists of three tiers that perform Primary Health Care (PHC) functions. The second level includes institutions such as departmental hospitals and specialized medical offices. The third level comprises university and specialized hospital (23). It is a public-private system, with a predominant public sector that is not necessarily statistical but, in terms of governance, is a system managed by the state (1).

Primary Health Care (PHC), according to the PES, includes services such as maternal and child health, control of communicable and non-communicable diseases, nutrition, mental health, dental care, and emergency services. In Haiti, PHC faces challenges such as a shortage of human resources, insufficient funding, and limited coverage. (24) classifies Health Centers (CCS) and Health Posts (CS) among the least efficient in the healthcare system.

CCS, the first entry point for PHC, provides essential services, including early disease screening (23). Predominantly located in rural areas, they serve populations of 5,000–6,000, divided into smaller sectors of about 1,000 inhabitants each. The PES aims to establish a CCS in every communal section, totaling 571 units. The Multipurpose Community Health Workers (ASCPs) and Nursing Assistants (AIs) are the key professionals in these centers. ASCPs promote hygiene, vaccination, and disease screening (e.g., malaria and HIV/AIDS), while AIs, with 1–2 years of technical training, oversee their work.

CS, the second level of PHC, serve populations of 25,000–30,000, offering curative, preventive, and health promotion services. Located mainly in semi-urban areas, they complement CCS and employ general practitioners and midwife nurses. Community Referral Hospitals (HCR), the third level of PHC, cover populations of 250,000–300,000, providing complex first-line care but offering direct patient services only in exceptional cases.

At the secondary level, Departmental Hospitals (HD) provide specialized care, including trauma services, oncology, and dialysis, supporting PHC. At the tertiary level, University Hospitals (HU) integrate patient care, training, and research, offering highly complex treatments.

The 2017–2018 Health Service Delivery Assessment (EPSS) identified 1,048 healthcare facilities in Haiti: 34.16% public, 18% mixed, 17.28% private non-profit, and 29.79% private for-profit (25). The West Department has the highest concentration of facilities due to its high population density, while other departments maintain a relatively balanced distribution of health services.

**Figure 2:**
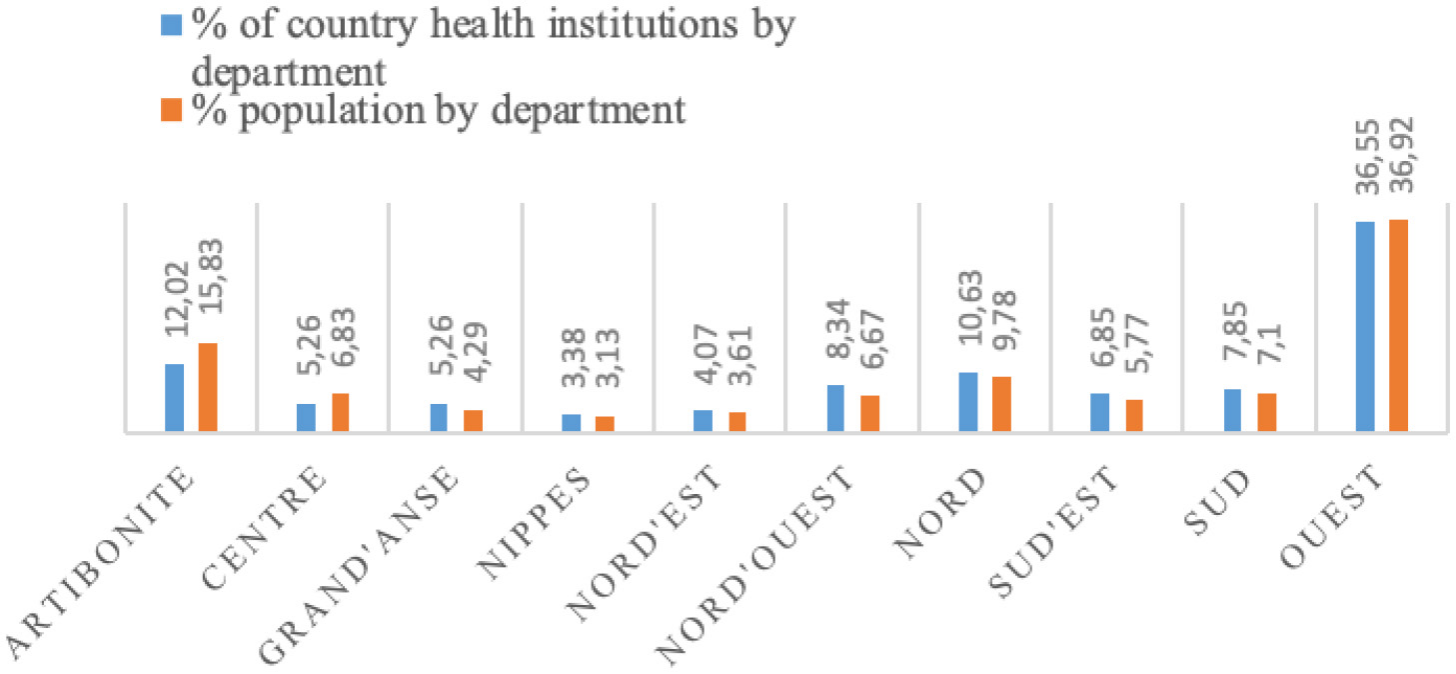
Haiti. Distribution of healthcare institutions and population by department (%) in 2021. Source: own elaboration based on Ref. (11).

The highest proportion of healthcare institutions was observed in the Ouest department, with 36.35%, followed by Artibonite with 12.02% and Nord with 10.63% (11). Nevertheless, Artibonite, one of the largest departments in Haiti, remains the one with the lowest proportion of healthcare facilities relative to its population, exhibiting the greatest disparity. Predominantly rural, 57.2% of its population lives in rural areas, compared to only 15.7% in the Ouest department (26). Despite its high population density, access to healthcare services remains limited due to the insufficient number of medical facilities, as illustrated in Figure 2.

Another factor that may explain these figures is the rise in violence and territorial control. In recent years, increasing violence and the occupation of territories by armed groups, particularly in the metropolitan area and the Artibonite department (27), have hindered the population's access to healthcare services and disrupted the operation of various medical units. These factors represent key causes of the low healthcare coverage in relation to the population of this department.

The private sector plays a significant role in healthcare service management, overseeing nearly half of the institutions, with 29.79% being for-profit and 17.28% non-profit. Meanwhile, the public and mixed sectors account for 34 and 19%, respectively. The healthcare system's high dependence on the private sector may impact accessibility, equity, and efficiency nationwide. It is well-known that private services tend to be more expensive and are concentrated in urban areas in Haiti. This not only limits access for low-income populations but also leaves those living in rural areas without access to basic healthcare services.

**Figure 3:**
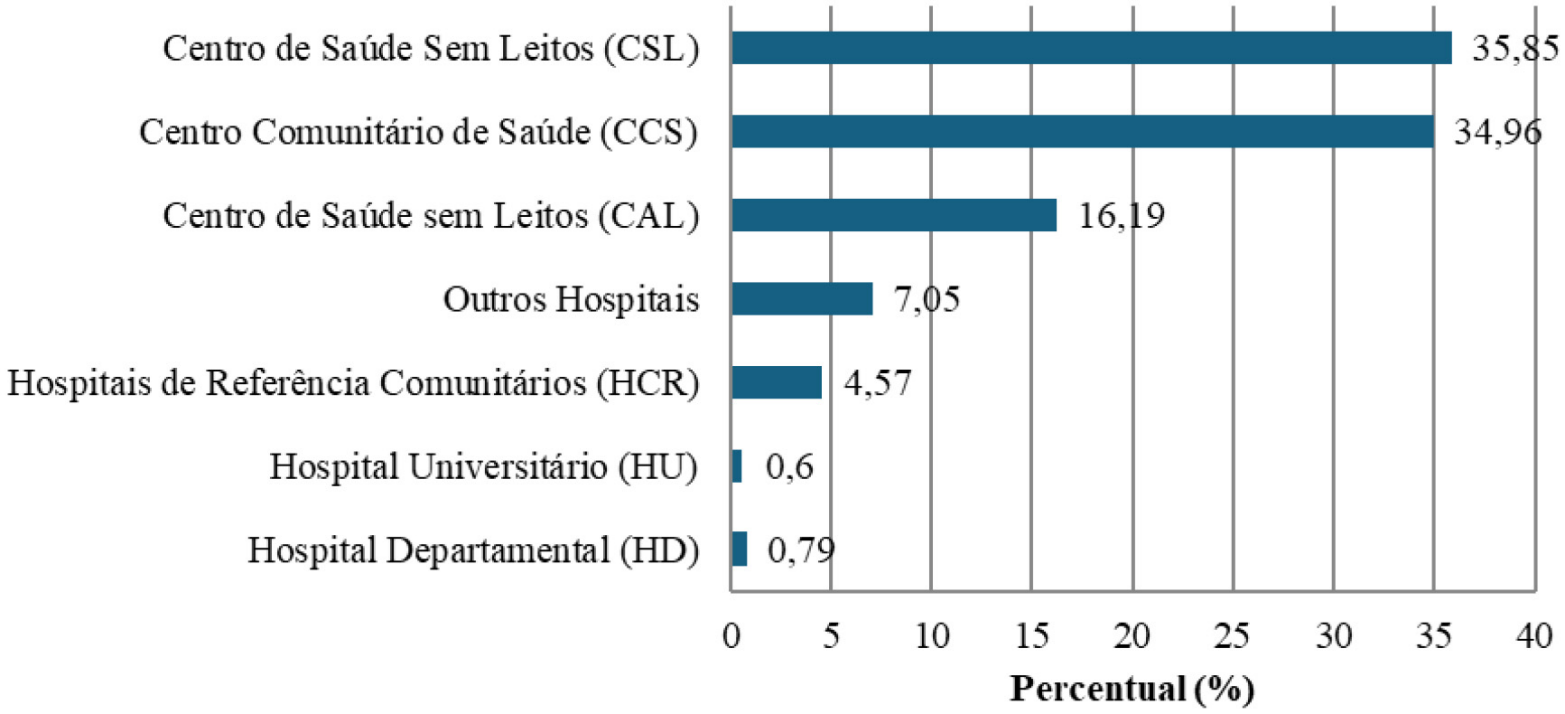
Distribution of healthcare institutions in the country by category. Figure created by the authors based on Ref. (11), own translation.

Figure 3 presents the distribution of healthcare institutions in the country by category, where the CCS, CSL, and CAL, which constitute the first tier of primary healthcare, collectively represent more than 80% of healthcare institutions.

According to the PES, Health Centers without Beds (CSL) and Health Centers with Beds (CAL) are collectively referred to under the generic designation of CCS. Table 1 presents the distribution of healthcare institutions by department and category in Haiti.

**Table 1:** Distribution of healthcare institutions by department and category in Haiti.

**Departamento**	**Inhabitants**	**% Population**	**CCS (%)**	CSL^*^(%)	**CAL (%)**	**Hospital (%)**
Artibonite	1.881.984	15.83	21.43	5.51	10.88	8.20
Center	812.958	6.83	7.83	4.06	8.16	3.28
Grand Anse	510.172	4.29	8.29	2.61	3.40	2.46
Nippes	373.151	3.13	4.61	0.58	4.76	2.46
Nord	1.162.594	9.78	8.76	12.75	7.48	10.66
Nord'Est	429.192	3.61	5.53	2.32	5.44	1.64
Nord'Ouest	793.970	6.59	15.90	2.32	8.16	3.28
Ouest	4.390.005	36.92	12.44	59.71	36.05	57.38
Sud	844.262	7.10	8.76	4.35	9.52	8.20
Sud'Est	686.163	5.77	6.45	5.80	6.12	2.46
Total	11.887.456	100.00	100.00	100.00	100.00	100.00
			434	345	147	122
Health Centers without Beds (CSL)						
Health Centers with Beds (CAL)						

Source: Ref. (28).

There is a concentration of healthcare institutions in the Ouest department, where the country's capital is located. This department accounts for 57.38% of hospitals, 59.71% of health centers without beds, 36.05% of health centers with beds, and, to a lesser extent, 12.44% of CCS facilities. On the other hand, the departments of Artibonite (21.43%) and Nord-Ouest (15.90%) show a higher prevalence of CCS facilities. Notably, after Ouest, the Nord department has the second-highest number of hospital centers, accounting for 10.66% of the total (28).

The Ouest department, which is predominantly urban, exhibits a higher demand for healthcare services. According to EMMUS VI 2016–2017 (29), the utilization of healthcare facilities is more frequent in urban areas (84%) than in rural areas (77%), reinforcing the need for greater service availability in rural regions. Additionally, international organizations operating in the healthcare sector have concentrated their activities in this department. According to (30), Ouest has also benefited from targeted investments, both through government policies and NGO projects, which have contributed to improvements in infrastructure and working conditions within the local healthcare system.

In other departments, such as Nord-Est, Sud-Est, Nippes, and others, where economic activity is lower, rural areas are predominant, and poverty levels are higher, the distribution of healthcare institutions is significantly lower. Consequently, the redistribution of healthcare institutions in Haiti can be attributed to various factors, including economic, geographical, political, and social aspects that do not support equitable access to healthcare services.

One of the main issues in Haiti's healthcare system is financing. According to data from the World Bank (12), Haiti has one of the lowest per capita public health budgets in Latin America and is increasingly dependent on international aid and NGO contributions. Although the National Health Policy (PNS) and the Health Master Plan (PDS) 2012–2022 established guidelines for a National Health Fund, financing strategies have not yet been formally defined (21).

The PNS (2012), developed in response to the humanitarian crisis caused by the 2010 earthquake during the administration of Joseph Michel Martelly (2011–2016) and led by Minister Dr. Alex Larsen, is based on the principles of universality, equity, comprehensiveness, and quality. The PNS sets strategic objectives such as expanding healthcare coverage, strengthening the role of the MSPP, and ensuring sustainable financing for the sector. The main areas of intervention include infrastructure expansion, human resource management, medication supply, and performance-based financing, supported by other ministries and partners for an integrated approach.

Funding comes from the public treasury, taxes on harmful products, and international support. To structure the implementation of the National Health Policy (PNS), the MSPP developed the Health Master Plan (PDS) 2012–2022, aligned with national and international guidelines, including the Millennium Development Goals (MDGs). Published in 2013, under President Joseph Martelly and the leadership of Dr. Florence D. Guillaume, the PDS was developed in collaboration with national and international partners. The plan aims to expand healthcare access, reduce morbidity and mortality, and strengthen governance, prioritizing decentralization, infrastructure, human resources, and sustainable financing.

Despite these guidelines, health remained a low political priority, leading to insufficient funding. In 2020, only 3.22% of GDP was allocated to health, below the average of comparable countries (12). Per capita health expenditure declined from $59.8 (2017) to $51.5 (2019), compared to $491 in the Dominican Republic and $949 in Latin America (31, 32). Currency depreciation further increased the costs of importing medicines and equipment, with the exchange rate rising from $64.77 (2017) to $88.81 (2019) (33).

Public health funding in Haiti comes from current revenues and donations (34), including domestic and customs taxes. Donations are provided through budget support and project funding, while loans and treasury bonds supplement resources. Health insurance, regulated by OFATMA, requires a 6% contribution from gross salary, shared between employer and employee (35). Health expenditures fluctuated: 3.75% of GDP under Aristide (2001–2004), 4.81% under Martelly (2011–2016), and 3.22% under Moïse (2017–2021), reflecting an overall decline. The PDS target of allocating 15% of the national budget was not met.

Health expenditures in Haiti (3.22% of GDP) remain below the average for low-income countries (5.41%) and Latin America and the Caribbean [8.63%; World Bank, 2024 (12)]. The share of the national budget allocated to health continues to decline, moving further away from the WHO recommendation (9).

According to (36, 37), public health funding in Haiti has significantly declined, while external aid has increased. Between 1995 and 2019, the share of public health expenditure in total health spending dropped from 1 to 9.68%, whereas external funding peaked at 70% of total health spending in 2011 before falling to 44.45%. This dependence makes the system vulnerable to fluctuations in international funding (37).

The PNS 2012–2022 indicates that health expenditures account for 3.3% of household income, yet only 9% of rural families, compared to 28% in metropolitan areas, believe they can afford healthcare costs. In a country where 80% of households face food insecurity, access to healthcare remains limited (38).

With a per capita expenditure of $44.18 in 2020 (12), Haiti should have achieved better health outcomes. However, reliance on out-of-pocket payments exacerbates inequalities. Many patients delay or avoid care due to costs, impacting system equity and efficiency (38). Between 2009 and 2010, 80% of the $75 million health budget was allocated to salaries, leaving limited funds for services. Health financing in Haiti remains a major challenge, requiring greater government commitment and diversified funding sources.

According to (39), over 140 million people in Latin America and the Caribbean lack access to healthcare, with 121 million affected by poverty and 107 million living in remote areas. Despite the legal recognition of the right to health, exclusion varies by country. In Mexico, Chile, and Cuba, fewer than 10% of the population face difficulties, whereas in El Salvador, Bolivia, Paraguay, and Haiti, more than 50% express dissatisfaction (39). In Haiti, births attended by qualified health professionals increased from 3% in 2017 to 75.8% in 2020 (36).

In recent years, Haiti has integrated traditional midwives (“matronas”) into the formal healthcare system, providing training and encouraging them to refer pregnant women to health facilities, alongside awareness campaigns promoting assisted childbirth (24, 40). Despite these efforts, maternal hospital mortality stood at 168.2 per 100,000 births in, 2021 and neonatal mortality at 25 per 1,000 live (36). In comparison, maternal mortality in the Dominican Republic was 169, and in Brazil, 107.53 (17, 41).

According to (42), 94 out of 100 Haitian children survived until age five, while only 78% of 15-year-olds lived to 60 due to limited healthcare access. Life expectancy at birth was 63.19 years in 2021 (33).

Primary healthcare (PHC) coverage in Haiti is assessed by the number of facilities providing these services, with only 42% of health institutions meeting the criteria (25). Limited PHC coverage affects essential services like vaccination and prenatal care, increasing the burden on secondary and tertiary care. In 2023, 23.5% of the population sought outpatient consultations, a 2.17% increase from 2020 (Table 2).

**Table 2:** Percentage of the population with access to basic healthcare services by department in Haiti in 2023.

**Department**	**Population**	**Number of first visits**	**Percentage of the population who accessed services**
Artibonite	1.928.565	422.343	21.9
Center	833.079	307.383	36.9
Grand'Anse	522.800	144.678	27.7
Nippes	382.386	96.029	25.1
Nord	1.191.370	319.727	26.9
Nord	439.815	106.786	24.3
Nord' Ouest	813.622	193.522	23.8
Ouest	4.498.663	795.701	17.7
Sud	865.164	312.128	36.1
Sud ‘Est	706.220	135.168	19.1
Total	12.181.686	2.833.465	23.5

Source: Ref. (22), own translation.

Henrys (9) observed a decline in healthcare attendance rates in Haiti, from 0% in 2016 to 19% in 2021. This decrease reflects growing access restrictions due to inadequate infrastructure, shortages of healthcare professionals, and limited financial resources, particularly affecting rural and impoverished areas. The MSPP acknowledges that the healthcare system is fragmented, poorly accessible, and of low quality.

Socioeconomic inequality and the ongoing political and security crisis exacerbate the situation, hindering healthcare services and restricting access. Many individuals' resort to informal alternatives due to distance, insecurity, or high costs.

Although the total number of healthcare users has decreased, visits per patient have increased, suggesting that a specific segment of the population—those with financial means or better access—continues to seek medical care regularly. This concentration of visits highlights the lack of universal coverage. Organizations such as Médecins Sans Frontières (MSF) primarily operate in the Western Department due to armed group blockades, further limiting access to other regions (43).

In Haiti, the number of healthcare professionals is insufficient, with the highest coverage in the Western region, where Port-au-Prince has 1.31 professionals per 1,000 inhabitants (Table 3). In, 2022 the country had only 0.28 physicians per 1,000 inhabitants, compared to 2.23 in the Dominican Republic and 2.14 in Brazil—far below the WHO standard of 25 healthcare professionals per 1,000 (44).

**Table 3:** Distribution of total healthcare professionals by department according to WHO criteria in Haiti in 2022.

**Department**	**Population**	**Doctors/ 1,000 Inh**.	***Matronas/* 1,000 Inh**.	**Nurses/ 1,000 Inh**.	**Aid for nurses/ 1,000 Inh**.	**Medical team Total/ 1,000 Inh**.	**Health personnel/ 1,000 Inh**.
Artibonite	1.881.984	0.12	0.005	0.24	0.27	0.63	1
Centro	812.958	0.14	0.007	0.22	0.36	0.72	1
Grande Anse	510.172	0.12	0.001	0.35	0.20	0.66	1
Nippes	373.151	0.18	0.005	0.26	0.20	0.64	1
Norte	1.162.594	0.32	0.006	0.56	0.22	1.11	1
Nord'Est	429.192	0.18	0.003	0.38	0.30	0.86	1
Nord'Ouest	793.970	0.1	0.006	0.25	0.30	0.64	0
Ouest	4.390.005	0.46	0.04	0.55	0.26	1.31	0
Sud	844.267	0.25	0.01	0.58	0.36	1.16	7
Sud'Est	689.163	0.18	0.007	0.32	0.16	0.67	1
Total	11.887.456	0.28	0.01	0.05	0.26	0.99	1

Source: Ref. (22), own translation.

Additionally, 60% of healthcare personnel work in hospitals, while only 8% serve in community health centers, contradicting the MSPP's policy (9). This imbalance weakens Primary Health Care (PHC), which could address up to 95% of health issues but lacks qualified professionals.

Human Resources for Health (HRH) priorities in Haiti are outlined in key MSPP documents, including the Health Master Plan (2012–2022, 2021–2031) and the Strategic HRH Development Plan 2030. However, these documents provide only general guidelines, lacking concrete policies and interventions (21).

The massive migration of healthcare professionals to the U.S., Canada, and Brazil exacerbates workforce shortages, particularly in the Center, Artibonite, and North departments. Haiti has approximately 10 public institutions and over eighty private universities offering health-related programs (22).

Midwifery training is available only in the North, South, and West, with programs at the Henry Christophe Campus (UEH Limonade), in Cayes, and in Port-au-Prince (45, 46). Despite advancements in training, limited availability restricts equitable access to reproductive health education.

## Final considerations

The Haitian healthcare system faces significant structural challenges that hinder equitable access to services for a substantial portion of the population. Over the past decade, under the administrations of Joseph M. Martelly, Jocelerme Privert, and Jovenel Moïse, important initiatives have been implemented, including the formulation of various strategic plans. While these actions have clearly outlined the MSPP's guidelines through, 2030 the objectives remain far from being achieved.

The Haitian healthcare system's reliance on external aid, combined with declining public investment in the sector, inadequate infrastructure, a shortage of human resources, poor organization and management, as well as political and security instability, has had profound impacts on the sector.

The distribution of human resources often contradicts the principles established by the MSPP, undermining equity in service access. Dependence on external aid weakens the system's autonomy, leading to fragmented interventions and priorities dictated by external donors, which are often disconnected from local needs. The predominance of the private sector, including non-governmental organizations (NGOs), in the healthcare system further exacerbates inequalities, particularly in access to services. These factors underscore the urgent need for investments in infrastructure, training, and the redistribution of human resources, along with more efficient governance, to ensure equitable and sustainable access to healthcare.

The challenges faced by the Haitian healthcare system are complex and multifaceted. It is of utmost importance that the MSPP commits to implementing strategic reforms to effectively strengthen primary healthcare coverage and improve equitable access to basic services, especially in remote areas of the country. In the current context of state and institutional crisis faced by the country, the implementation of health policies encounters significant obstacles. However, prior to any concrete action, it is crucial that there be political will on the part of government authorities. The challenges facing the health system are complex and multifaceted, requiring approaches that consider multiple dimensions. In this scenario, it is recommended that the State adopt an alternative financing model based on public-private partnerships (PPPs). Such partnerships can support the financing of infrastructure, hospital management, and the provision of essential services, as observed in several Latin American countries. To ensure effectiveness and prevent service fragmentation, it is necessary to establish coordination committees that bring together the Ministry of Health, non-governmental organizations, and international actors.

It is important to note that the data used in this article is primarily drawn from the MSPP, which is considered the official source of information on the Haitian healthcare system. However, when these data are compared with those from independent sources, such as national and international NGOs, significant discrepancies may be observed. It must be acknowledged that certain healthcare institutions in the country often operate with distinct resources, treatment protocols, and patient populations, which may influence the nature and representativeness of the findings presented in this article.

These discrepancies can be attributed, in part, to information gaps or the limited oversight of private institutions and NGOs operating within Haiti's health sector. This divergence represents a significant limitation in the interpretation of the results, as it highlights the complexity and fragmentation of the national healthcare system. Nonetheless, this article makes a valuable contribution to the understanding of how the Haitian health system functions, particularly by underscoring its growing dependence on external aid, the insufficiency of infrastructure, the shortage of qualified human resources, and persistent issues related to organization and governance. Consequently, one of the main limitations of this study is the lack of primary data on the coverage of the Haitian health system, which hinders a more precise analysis of the impact of existing policies and the conduct of a cross-analysis between secondary and primary data.

## Data Availability

The original contributions presented in the study are included in the article/supplementary material, further inquiries can be directed to the corresponding authors.
